# First Principles of Otology

**Published:** 1904-01

**Authors:** 


First Principles of Otology. A Textbook for Medical Students. By Albert H. Buck, M. D. Clinical Professor of the Diseases of the Ear, College of Physicians and Surgeons, Medical Department of Columbia University, New York. Duodecimo, pages 227. Second edition. Illustrated. New York: William Wood & Company. 1903. (Price, $1.50.)

The book before us is one of the few good ones that help to fill the gap between the treatise and the quiz-compend. It opens with a brief recitation of the methods of examination of the external, middle and internal ear, and then takes up the different structures in regular order,—auricle, external canal, middle ear and mastoid process and internal ear. At the beginning of each subject is a short paragraph on anatomical considerations, evidently intended only to refresh the memory, the author assuming that the reader knows the anatomy; and this is followed by a few tersely worded generalities under the heading of pathology. The diseases described are the commonplace ones, that are familiar to every practitioner, and no space is wasted upon the unusual in any of its phrases. Although everything of importance is touched upon, brevity is one of the objects of the book, and is very well carried out.

The chapter on catarrhal middle ear affections, deals with the throat in its relation to these diseases, laying stress upon adenoid vegetations in the nasopharynx. Extradural abscess, infective thrombosis of the sigmoid sinuses and abscess of the brain are included in the chapter on mastoid disease, and the other chapters are equally comprehensive. A feature of the volume is the grouping together under one head of the pathological attractions of the membrana tympani and their significance. The book proper ends with diseases of the internal ear and auditory nerve, but there is an appendix in two sections devoted to the anatomy and physiology of the whole organ of hearing, the first section covering the middle ear and the second the labyrinth. Both are discussed rather exhaustively.

The book is very convenient in size (duodecimo) and teems with good points, which are set forth in clean-cut, vigorous English, abounds in simple facts, clearly stated, and is an altogether admirable work. L. B., Jr.

